# The role of vitamin D signalling in β cell apoptosis: a systematic review

**DOI:** 10.1007/s10495-026-02453-5

**Published:** 2026-10-01

**Authors:** Donglai Li, Nick Norris, Yousun An, Jenny E. Gunton

**Affiliations:** 1 Faculty of Medicine & Health, University of Sydney, Camperdown, Sydney, NSW 2050 Australia; 2 Centre for Diabetes, Obesity and Endocrinology Research, The Westmead Institute for Medical Research, Westmead, Sydney, NSW 2145 Australia; 3 Department of Diabetes and Endocrinology, Westmead Hospital, Westmead, Sydney, NSW 2145 Australia

**Keywords:** Vitamin D, Vitamin D receptor, Apoptosis, Beta cell, Diabetes

## Abstract

**Background:**

Vitamin D signalling, primarily through the vitamin D receptor (VDR), regulates cellular survival and programmed cell death. This systematic review aims to synthesise current evidence on the role of vitamin D in pancreatic β-cell apoptosis.

**Methods:**

Medline, Embase, and Scopus were searched for original studies investigating vitamin D, vitamin D analogues, or VDR-related interventions in experimental models of β-cell apoptosis or programmed cell death.

**Results:**

26 studies were included. Across in vitro, ex vivo, and in vivo models, vitamin D signalling protected β-cells against apoptosis induced by cytokine-mediated inflammation, oxidative stress, glucotoxicity, lipotoxicity, streptozotocin exposure, and diabetic stress. These effects were associated with reduced NF-κB signalling, nitric oxide and reactive oxygen species production, improved mitochondrial stability, modulation of Bcl-2 family proteins, inhibition of endoplasmic reticulum stress, and regulation of pyroptosis, ferroptosis, and autophagy-related survival mechanisms. VDR activation also supported β-cell identity, insulin secretion, and β-cell mass preservation. In contrast, calcitriol-associated growth inhibition and increased apoptosis were reported in βTC insulinoma models.

**Conclusion:**

Vitamin D signalling generally appears cytoprotective in β-cells exposed to inflammatory, oxidative, and metabolic stress. However, its effects on β-cell survival are model- and context-dependent, reflecting differences in cellular background, experimental stressors, and methods used to assess cell death.

**Supplementary Information:**

The online version contains supplementary material available at https://doi.org/10.1007/s10495-026-02453-5.

## Introduction

The study of Vitamin D has evolved remarkably since its 19th-century identification as the vital link between rickets and insufficient sunlight exposure [1–3]. Originally characterised as a nutrient essential for calcium homeostasis and skeletal health, the discovery of the Vitamin D Receptor (VDR) and its presence across nearly all human tissues fundamentally expanded the scope of vitamin D research [4]. Upon the binding of its active metabolite, calcitriol [1,25(OH)₂D], VDR interacts with a range of transcriptional co-regulators and cytosolic signalling pathways, demonstrating both genomic and non-genomic activities that regulate critical biological processes, including the modulation of immune responses and the regulation of cellular growth and differentiation [5–8].

**Fig. 1:**
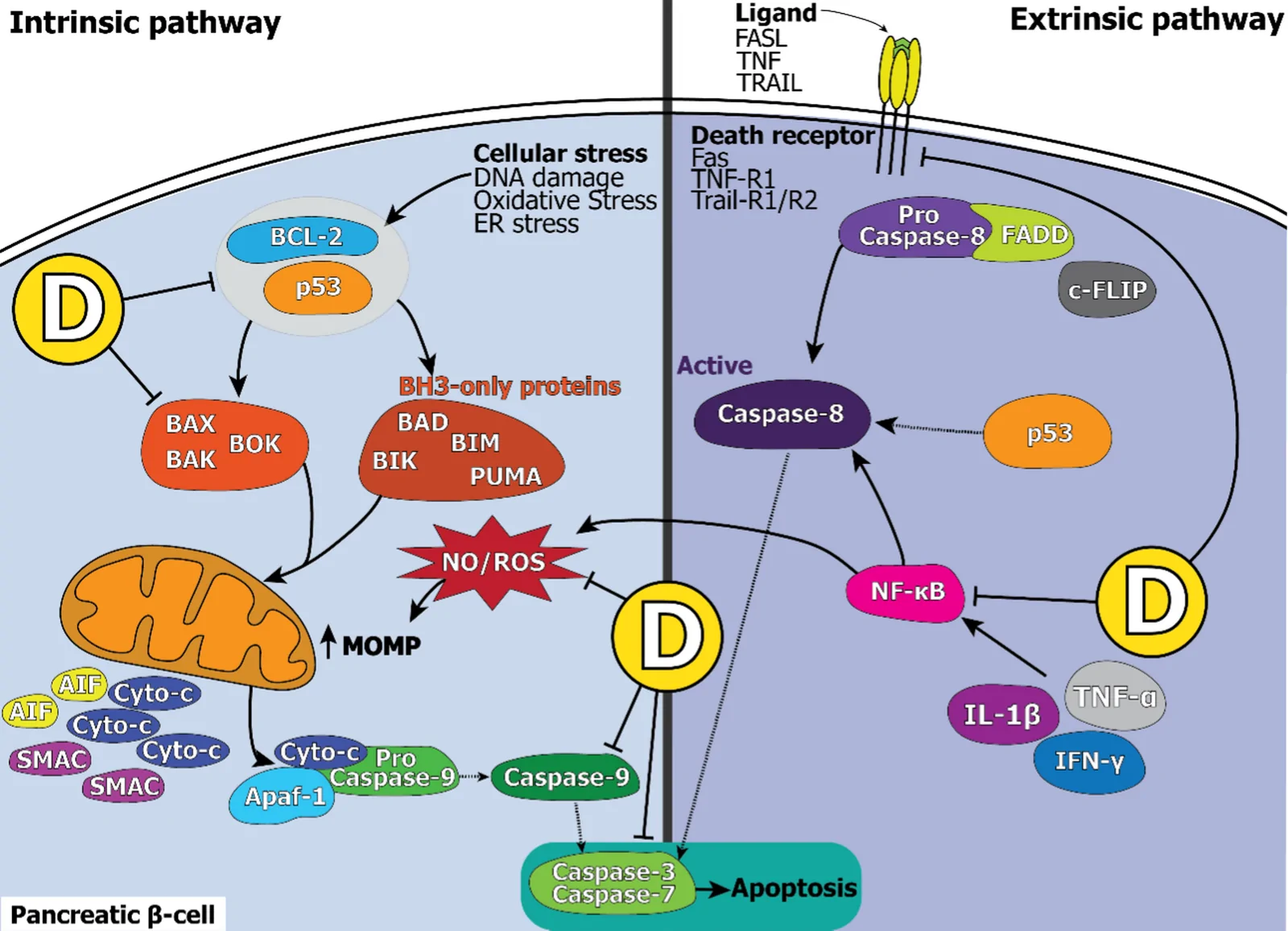
Vitamin D signalling modulates apoptotic pathways in pancreatic β-cellsCellular stressors, including DNA damage, oxidative stress, and ER stress, activate the intrinsic apoptotic pathway through p53-dependent regulation of BCL-2 family proteins. Activation of pro-apoptotic BAX/BAK and BH3-only proteins promotes MOMP, leading to cytochrome c release, apoptosome formation with Apaf-1, caspase−9 activation, and downstream activation of executioner caspases-3 and − 7. The extrinsic pathway is initiated by death ligands, including FASL, TNF, and TRAIL, which bind to their corresponding death receptors and promote FADD-mediated activation of caspase−8. Caspase−8 can directly activate caspase−3/7. Inflammatory cytokines, including IL-1β, TNF-α, and IFN-γ, activate NF-κB signalling, which contributes to β-cell apoptosis through increased inflammatory signalling, caspase−8 activation, and production of NO/ROS. NO/ROS further promotes mitochondrial dysfunction and MOMP. Vitamin D/VDR signalling, indicated by yellow “D” symbols, is proposed to protect stressed pancreatic β-cells by suppressing NF-κB activation, reducing NO/ROS production, limiting mitochondrial apoptotic signalling, and inhibiting downstream caspase activation.

Apoptosis is a fundamental, tightly regulated process of programmed cell death essential for maintaining tissue homeostasis and immune regulation. It is primarily executed through two interconnected pathways: intrinsic and extrinsic [9, 10]. The intrinsic pathway (Fig. 1) is triggered by internal stressors, such as DNA damage or oxidative stress, which activate B-cell lymphoma 2 (BCL-2) family proteins and the tumor suppressor p53, leading to mitochondrial outer membrane permeabilisation (MOMP) and the release of apoptogenic factors like cytochrome c [11–13]. This results in the formation of the apoptosome and the activation of caspase−9 [14]. Conversely, the extrinsic pathway (Fig. 1, right side) is initiated by extracellular ligands binding to death receptors (e.g., FAS (CD95/APO-1), tumour necrosis factor receptor 1 (TNF-R1), or TNF-related apoptosis-inducing ligand (TRAIL)), facilitating the assembly of the death-inducing signalling complex (DISC) and the activation of caspase−8 [15–17]. While the extrinsic pathway can directly activate executioner caspases-3 and − 7 in Type I cells, Type II cells including pancreatic β-cells—require an amplification step through the intrinsic machinery via MOMP [18, 19]. Ultimately, both pathways converge on the activation of executioner caspases to orchestrate systematic, controlled dismantling of the cell [10].

Dysregulation of programmed cell death disrupts physiological equilibrium and is implicated in the pathogenesis of many chronic conditions, including diabetes, cancer, autoimmune diseases, cardiovascular disorders, and neurodegenerative diseases [10, 20]. In diabetes mellitus, a key factor in the pathogenesis of both Type 1 (T1D) and Type 2 Diabetes (T2D) is the progressive reduction of functional β-cell mass [21, 22]. While T1D is primarily driven by autoimmune-mediated β-cell destruction and T2D by a combination of insulin resistance and progressive β-cell dysfunction, loss of functional β-cell mass is a common feature of both conditions [23, 24]. Apoptosis is an important contributor to β-cell loss; however, evidence indicates that β-cell death is heterogeneous and involves necrosis and other regulated cell-death pathways depending on the cellular stressors and experimental models [25–27].

In recent decades, research has demonstrated the roles of Vitamin D in regulating apoptosis and modulating cell survival across different cell types and physiological contexts. Beyond its classical influences, 1,25(OH)₂D exerts anti-apoptotic effects on osteoblasts and healthy chondrocytes [28–30], yet it selectively induces death in hypertrophic chondrocytes and hyperproliferative rheumatoid synoviocytes [31–33]. In various malignancies, including breast, colorectal, prostate, and lung carcinomas, Vitamin D and its analogues act as pro-apoptotic agents by upregulating tumor suppressors and pro-apoptotic proteins [34–41]. Furthermore, vitamin D signalling acts as a context-dependent immunomodulator in both innate and adaptive immune systems. 1,25(OH)₂D modulates dendritic cell maturation and activation, B-cell proliferation and differentiation, and T-cell responses [42–44]. In different cellular and inflammatory context, vitamin D inhibits apoptosis in *Mycobacterium tuberculosis*-infected THP-1 cells [45], whereas it promotes neutrophil apoptosis in patients with chronic obstructive pulmonary disease [46]. In non-obese diabetic (NOD) mice, 1,25(OH)₂D restores thymocyte sensitivity to apoptotic signals through coordinated effects on thymic T cells and dendritic cells, potentially improving thymic negative selection of autoreactive T cells [47]. The proposed role of vitamin D signalling of apoptosis in stressed pancreatic β-cell is illustrated in Fig. 1.

Given that epidemiological data associate Vitamin D deficiency with the pathogenesis of diabetes mellitus [48, 49], and that the depletion of functional β-cell mass through programmed cell death is a hallmark of both T1D and T2D [23, 24], this systematic review explores the involvement of Vitamin D in these survival pathways to elucidate its potential as a therapeutic mediator.

## Methods

A comprehensive review of the literature was conducted following the guidelines established by the Preferred Reporting Items for Systematic Reviews and Meta-Analyses (PRISMA) [50]. The PRISMA checklist is presented in Supplementary Materials Table 2. This review is registered with PROSPERO with the identifier CRD420251265631.

### Eligibility criteria

This systematic review included all primary research investigating the role of vitamin D or VDR signalling, whether through dietary intake, supplementation, or genetic modification, to assess their impact on apoptosis in pancreatic β-cells. Eligible studies were limited to those published in English and could include any type of study, both preclinical (in vivo or in vitro) and any form of trial. For human islet studies, no restriction was applied according to geographical location, season of tissue collection, or presumed sunlight exposure of donors.

Secondary literature was excluded from this review, including reviews, conference abstracts, editorials, protocols, and letters. Studies that did not evaluate pancreatic β-cells, did not employ vitamin D or VDR as an intervention or variable, or did not measure apoptosis or programmed cell death as an outcome were also excluded. Furthermore, reports were excluded if the full-text version could not be retrieved despite exhaustive searching and attempted author contact.

### Search strategy

We comprehensively searched Medline, Embase, and Scopus databases from their inception to December 2025, with the search executed on 12 December 2025. Our search strategy employed a combination of controlled vocabulary, including Medical Subject Headings (MeSH) and Emtree terms, such as 'Vitamin D' (e.g., cholecalciferol, calcitriol, or Vitamin D receptor), 'Apoptosis' (e.g., programmed cell death), and 'Beta cells' (e.g., insulin-secreting cells or islets of Langerhans). These terms were systematically combined to identify the intersection of the three concepts. The complete search strategies are detailed in the Supplementary Materials Table 1.

### Selection process

Following the initial search, all identified records were imported to EndNote 20 (Clarivate Analytics, Philadelphia, PA, USA) for duplicate removal and management. The selection process was divided into two separate stages. First, two reviewers, D.L. and J.G., independently evaluated the titles and abstracts of all records based on the established inclusion and exclusion criteria. Second, the full-text versions of the remaining studies were retrieved and reviewed for eligibility by both reviewers. Any differences or disagreements regarding the inclusion of studies at either stage were resolved through consensus.

### Data collection

Data extraction was conducted by D.L.. Extracted data included study identification (author, year), experimental model (cell line, species/strain, and genetic modifications), type of cellular stressor applied, and parameters of vitamin D intervention. The primary outcomes for this review focused on apoptosis and cell death-related findings, including both quantitative and qualitative changes in cell viability, proliferation, and programmed cell death, which encompassed markers such as caspase−3 activity, TUNEL staining, DNA fragmentation, and lactate dehydrogenase (LDH) release. Secondary outcomes addressed inflammation, oxidative stress outcomes, insulin release and secretion, and autophagy, as well as key signalling molecules and pathways implicated in the vitamin D responses. The data were categorised based on study characteristics, such as study model, type of stressor, type of vitamin D intervention, and apoptosis-related findings. Any discrepancies in the extracted data were resolved through team consensus and a secondary review of the original manuscripts.

### Risk of bias assessment

Risk of bias and methodological quality were assessed independently by D.L. and J.G. The SYRCLE's risk of bias tool was employed for animal-based in vivo studies [51]. Each reviewer evaluated the domains such as selection, performance, detection, attrition, and reporting biases. For cell-based in vitro experiments, adapted criteria were utilized. For both study types, each domain was categorized as having a 'Low,' ‘Moderate,’ 'High,' or 'Unclear' risk of bias. Discrepancies between reviewers were resolved through consensus or by consultation with a third reviewer (N.N. or Y.A.). The assessment report can be found in the Supplementary Materials Table 3 and 4.

## Results

### Search results

**Fig. 2:**
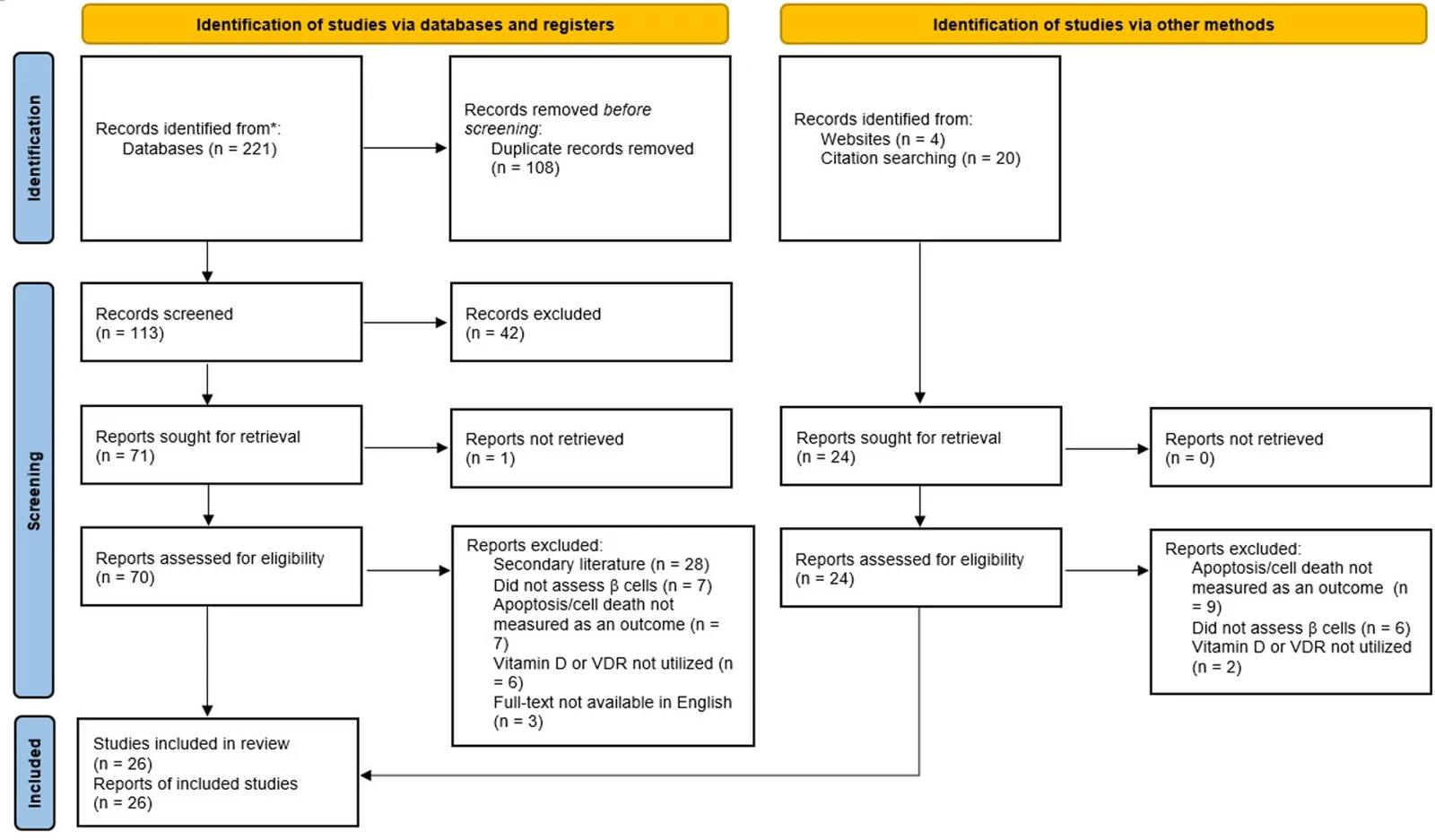
PRISMA flow diagram of literature search, screening, and selection process

We identified 221 articles through database searches and an additional 24 studies via alternative methods, including websites and citation exploration. Following the removal of duplicates, 113 records remained for screening, of which 42 were excluded based on the initial evaluation of titles and abstracts. From all methods combined, 94 out of 95 reports were successfully retrieved and assessed for eligibility. 68 studies were excluded for the following reasons: secondary literature (n = 28), lack of apoptosis/cell death measurement as an outcome (n = 16), absence of β cell evaluation (n = 13), non-utilisation of vitamin D or VDR as an intervention or variable (n = 8), and non-English full-text publication (n = 3). As a result, 26 studies were included in this review. 15 utilised a single-model approach (7 in vivo, 7 in vitro, and 1 ex vivo). The remaining 11 studies employed multi-model designs: 6 combined in vitro and in vivo, 2 combined in vitro and ex vivo, and 3 utilised all three methods. The process is illustrated in the PRISMA flow diagram (Fig. 2).

### In vitro and ex vivo studies

#### βTC/insulinoma models

The βTC3 cell line was derived from an insulinoma arising in a transgenic mouse model expressing SV40 T-antigen in pancreatic β-cells [52]. Using this model, Galbiati and researchers demonstrated that treatment with 1,25(OH)₂D inhibited cell proliferation and increased cell death in a dose-dependent manner. The authors attributed this primarily to apoptosis, supported by DNA fragmentation, nuclear condensation, and increased caspase−3 activity, while the absence of increased LDH release argued against substantial membrane-disruptive necrosis. This effect is mediated by a nongenomic activation of the mitogen-activated protein kinase (MAPK) pathway, specifically the extracellular signal-regulated kinase (ERK)1/2 proteins, which are activated by protein kinase C (PKC) through calcitriol [53].

In a subsequent study, Galbiati et al. investigated the molecular pathways associated with calcitriol-induced cell death and growth inhibition using p53-knockout and Bcl-2-overexpressing βTC models [54]. Calcitriol-induced loss of viability was strongly dependent on p53 status and resulted in a dose-dependent increase in p21, activated Bak and Bax, initiated growth arrest and DNA damage-inducible 45 (GADD45), and consequently led to apoptosis and growth inhibition. Notably, calcitriol induced a biphasic increase in NF‑κB, along with the induction of insulin-like growth factor (IGF)− 1 and suppression of IGF-II, which was proposed as a pro-survival adaptation of tumour cells [54].

Contrary to the findings in βTC3 cells, primary human benign insulinoma cells examined in the same 2002 study showed that calcitriol did not induce apoptosis [53]. Normal human islets demonstrated a significant decrease in insulin expression, content, and basal release with 20 min of calcitriol. This was mediated via a rapid, nongenomic response by increasing ERK1/2 phosphorylation. Apoptosis was not reported in primary human islets [53].

#### Cytokine-induced inflammatory stress

Cytokine-induced inflammatory stress is one of the most widely used experimental tools for studying β-cell apoptosis [55]. RINm5F β-cells, an insulin-secreting rat insulinoma-derived β-cell line, were treated with a pro-inflammatory cytokine cocktail containing IFNγ, IL1β, and TNFα to induce apoptosis [56]. Co-treatment with 1,25(OH)₂D reduced cytokine-induced apoptosis by approximately 40–45%, as assessed by the proportion of sub-G1 cells. In addition, 1,25(OH)₂D reduced loss of mitochondrial transmembrane potential, decreased nitric oxide (NO) production, and partially restored insulin secretion and metabolic activity. These effects were mediated through the upregulation and maintenance of the antiapoptotic protein A20/TNFAIP3 and the inhibition of NF‑κB activation and translocation to the nucleus [56].

In human islets, 1,25(OH)_2_D significantly reduced cytokine-induced apoptosis, reducing the apoptotic cell proportion by approximately 43% and 40% relative to cytokine treatment alone at 10 and 1000 nM, respectively. This was evidenced by reduced nuclear fragmentation and chromatin condensation. Pretreatment of 1,25(OH)_2_D also inhibited cytokine-induced IKKα/β activity; western analysis confirmed that this effect was associated with the up-regulation of the A20/TNFAIP3 protein [56]. The authors further reported that these protective effects are mediated through the suppression of the iNOS/NO pathway and the downregulation of the Fas receptor. Additionally, 1,25(OH)₂D modulated key inflammatory and apoptotic gene clusters, suppressing pro-apoptotic factors—including NF-κB p49/p100, caspase−8, second mitochondria-derived activator of caspases (SMAC), and apoptosis-inducing factor (AIF)—while restoring the expression of critical survival genes such as apoptosis inhibitor 3 (API3) and glucocorticoid-induced leucine zipper (GILZ) [57].

In contrast, pretreatment with 1,25(OH)₂D did not improve survival in INS-1E cells and FACS-purified rat pancreatic β-cells treated with IL-1β and IFN-γ, with no significant differences in either apoptotic or necrotic cell death between treatment conditions, despite reduced expression of several inflammatory and chemotactic genes, including IL-1β, MCP-1, IP-10, and IL-15 [58]. Importantly, cytokine-induced nitrite accumulation was unaffected by 1,25(OH)₂D, suggesting that suppression of inflammatory genes may have been insufficient to inhibit downstream cytotoxic signalling.

Wolden-Kirk and researchers reported that in human islets exposed to IL-1β and IFN-γ for 48 h, apoptosis increased from 1.0% to 11.0%, whereas 1,25(OH)₂D cotreatment reduced apoptosis to 2.0% [59]. In mouse islets, cytokine exposure increased apoptosis from 7.0% to 19.7% after 24 h, and 1,25(OH)₂D₃ partially reduced this to 12.6%. Longer cytokine exposure of mouse islets for 48 and 72 h produced a similar protective pattern, suggesting that the greater protection observed in human islets was not solely attributed by exposure duration. The proposed mechanism underlying the protection against apoptosis may involve delayed inhibition of NF-κB signalling. While 1,25(OH)₂D suppressed chemokine and cytokine expression early, its effect on apoptosis-related genes occurred at later time points. Likewise, more cytokine-altered genes were normalised by 1,25(OH)₂D at 24 h than at 6 h, supporting a time-dependent transcriptional response. 1,25(OH)₂D also restored glucose-stimulated insulin secretion (GSIS) in human islets from 72% of control levels after cytokine exposure to 99% of control levels, while fully restoring cytokine-impaired insulin secretion in mouse islets*.* However, the same treatment did not protect INS-1E cells from cytokine-induced cell death. The authors suggested that this discrepancy may reflect species- and model-dependent differences [59].

Wei et al. reported critical role of VDR in maintaining β-cell survival and function under stress using shRNA knockdown of VDR in human induced pluripotent stem (iPS) cell-derived β-like cells. They found VDR knockdown markedly increased cell vulnerability to IL-1β-induced apoptosis. VDR knockdown in INS1 cells led to a significant reduction in the expression of β-cell identity genes, including Ins2, Nkx6-1, Pdx1, and Ucn3, when challenged with cytokines or palmitic acid (PA) [60]. Prolonged IL-1β exposure dysregulated over 2,000 genes in isolated mouse islets. Co-treatment with Calcipotriol, a synthetic VDR ligand, partially reversed these effects by repressing inflammatory response genes and restoring those critical for β-cell identity, providing a dual-mechanism defense [60].

#### H₂O₂/cadmium-induced oxidative stress

Hydrogen peroxide (H_2_O_2_) or cadmium serve as a tool to mimic the oxidative stress environment often seen in diabetes [61, 62]. 1,25(OH)₂D has been demonstrated to enhance the viability of NIT-1 mouse insulinoma-derived β-cells following cadmium-induced damage by mitigating oxidative stress. During prolonged low-dose cadmium exposure, 1,25(OH)₂D also partially restored insulin secretion; however, it did not reverse the cadmium-associated reduction in insulin receptor substrate (IRS)− 1 and − 2 [63].

Chen et al. demonstrated that H_2_O_2_ upregulates FoxO1, which induced mitochondrial-dependent apoptosis in EndoC-βH1 human β-cells (derived by treating fetal pancreatic cells with lentiviral-SV40LT) [64]. VDR overexpression or calcitriol treatment promoted cell survival and mitigated forkhead box protein O1 (FoxO1)-induced mitochondrial dysfunction and apoptosis (~ 10%). Conversely, VDR silencing and mutant VDR resulted in a significant increase in FoxO1 expression and a decrease in cell survival [64].

In H_2_O_2_-treated MIN6 mouse insulinoma-derived β-cells, pretreatment with 1,25(OH)₂D enhanced cell viability from 61 to 85%. Consistently, the proportion of early apoptotic cells decreased from 28.2% to 9.32% [65]. This protective effect was accompanied by an increased Bcl-2/Bax ratio and decreased caspases activity, achieved through the inhibition of protein kinase RNA-like endoplasmic reticulum kinase (PERK) phosphorylation and downstream ATF4/CHOP expression [65].

#### STZ-induced cytotoxicity

He and researchers identified autophagy as a critical survival mechanism triggered by vitamin D in streptozotocin (STZ)-treated INS-1 cells. By significantly upregulating autophagy-related markers such as Beclin-1 and LC3-II, 1,25(OH)₂D reduced reactive oxygen species (ROS) and malondialdehyde levels with enhanced total antioxidant capacity [66].

Zheng et al. recently discovered an alternative protective mechanism by which vitamin D mitigates damage in STZ-treated MIN6 cells through the downregulation of the pyroptosis pathway. 1,25(OH)₂D treatment significantly suppressed the expression of NOD-like receptor protein 3 (NLRP3), NF-κB, caspase−1, and gasdermin D (GSDMD), while notably reducing the release of LDH, a key marker of cell membrane rupture [67].

#### Glucotoxic stress

A transcriptomic study conducted by Bornstedt and colleagues has identified several genes involved in apoptosis and cell survival that are targeted by 1,25(OH)₂D in glucose-challenged INS1E cells [68]. One such gene is SERPINA3 (increased by 1.36-fold), a member of the serine protease inhibitor superfamily, which has implication in apoptosis depending on the context and cell type [69–71]. The researchers also found that calcitriol reduced *Casp3* expression by 1.23-fold relative to glucose-stimulated control cells. Furthermore, 1,25(OH)₂D enhanced the expression of fibroblast growth factor (FGF), *Igf1*, and prolactin signalling pathways, potentially supporting β-cell viability and proliferation [68].

By activating the AMP-activated protein kinase (AMPK)/PGC-1α and suppressing mammalian target of rapamycin (mTOR) pathways, 1,25(OH)₂D has been shown to reverse the oxidative and inflammatory stress caused by high glucose exposure in INS-1E cells [72]. This shift leads to a significant reduction in ROS production and inhibits the TXNIP/NLRP3/IL-1β inflammasome axis. Consequently, Vitamin D treatment prevented cell death by inhibiting the activation of caspase−1 and caspase−8, reducing overall pyroptosis by 40.9% [72].

Similarly, Zhao and researchers showed that 1,25(OH)₂D acts as a potent protective agent by disrupting the NF-κB-DMT1 signalling axis in INS-1 cells exposed to high glucose. Vitamin D reduced inflammation, high glucose-induced upregulation of Ferritin Heavy Chain (FTH), and apoptosis by blocking the nuclear translocation of the NF-κB p65 subunit [73].

Beyond its anti-inflammatory effects, vitamin D prevented ferroptosis in high glucose-stressed INS-1 cells. The treatment significantly reduced ROS and intracellular iron accumulation by increasing protective enzyme glutathione peroxidase 4 (GPX4) and decreasing the pro-ferroptotic enzyme acyl-CoA synthetase long-chain family member 4 (ACSL4). Moreover, 1,25(OH)₂D improved cell survival in a VDR-dependent manner by reversing the upregulation of FOXO1 triggered by high glucose exposure [74].

#### Lipotoxic stress

In a study where palmitic acid (PA) was utilised as the primary stressor in MIN6 cells, Liu et al. demonstrated that 1,25(OH)₂D provided a dose-dependent reduction in pro-inflammatory markers such as TNF-α and IL-6, while simultaneously inhibiting apoptosis and decreasing elevated endoplasmic reticulum (ER) stress markers including CHOP and GRP78 [75]. These protective effects were accompanied by a significant reduction in ROS and malondialdehyde (MDA) levels, while enhancing the antioxidant defences such as glutathione (GSH) and superoxide dismutase (SOD) [75].

## Summary of in vitro and ex vivo studies

Cell-culture studies demonstrated heterogeneous responses to vitamin D signalling. Two studies from the same research group reported calcitriol-associated growth inhibition and apoptosis-related cell death in βTC insulinoma cells, whereas other insulinoma-derived β-cell lines showed neutral or cytoprotective responses when challenged with inflammatory, oxidative, or metabolic stress (Table 1). Under cytokine-induced inflammatory stress, 1,25(OH)₂D treatment reduced apoptosis, preserved mitochondrial potential, and restored insulin secretion in RINm5F cells by upregulating the anti-apoptotic protein A20 and blocking NF-κB translocation; though this protective effect was not mirrored in INS-1E cells. In models of diabetes-related oxidative stress induced by H_2_O_2_ or cadmium, 1,25(OH)₂D and VDR signalling consistently preserved β-cell viability in NIT-1 and EndoC-βH1 human β-cell lines, it counteracted mitochondrial dysfunction and apoptosis by downregulating FoxO1, inhibiting the PERK/ATF4/CHOP ER stress pathways, and shifting the Bcl-2/Bax ratio to suppress downstream caspase activity. Similarly, in models of STZ-induced cytotoxicity, 1,25(OH)₂D decreased cytotoxicity through two survival mechanisms: promoting autophagy and inhibiting pyroptosis. Finally, under conditions of metabolic overload, 1,25(OH)₂D protected β-cells from glucose-driven apoptosis, pyroptosis, and ferroptosis by promoting proliferation and survival signalling (FGF, IGF-1, prolactin, and SERPINA3), suppressing caspase activity through AMPK activation, and reducing inflammation by disrupting the NF-κB-DMT1 axis. Vitamin D provided dose-dependent protection against lipotoxicity by reducing ER stress and inflammatory cytokines, and boosting intrinsic antioxidant defenses.

**Table 1:** In vitro studies of vitamin D signalling on pancreatic β-cell apoptosis and survival

Study	Cell Model	Treatments	Outcomes
*βTC Insulinoma Cells*			
Galbiati et al. [53]	Murine insulinoma cell line (βTC3)	1,25(OH)_2_D at 10, 100, & 1000 nM for 48 and 72 h	↑ Apoptosis, ↓ Viability, ↓ Proliferation, ↓ Insulin, ↑ MAPK
Galbiati et al. [54]	Murine insulinoma cell line (βTC3, BTC (p53+/+), BTC (p53−/−), BTCtet, BTCtet/Bcl-2)	1,25(OH)_2_D at 10, 100, & 1000 nM for 20 min, and 4 to 48 h	↑ Apoptosis, ↑ Cell cycle arrest, ↓ Viability, ↑ p53, ↑ NF-κB, ↓ Bcl-2 & Bcl-xl
*Cytokine-induced inflammatory stress*			
Riachy et al. [56]	Rat β cell line (RINm5F)	1,25(OH)_2_D at 10 & 1000 nM concurrently with cytokine exposure for 48 h	↓ Apoptosis, ↑ Viability, ↑ Δψm, ↑ Insulin, ↓ NO, ↑ A20, ↓ NF-κB
Gysemans et al. [58]	Rat β‑cell line (INS-1E)	1,25(OH)_2_D at 10 nM for 24 h before cytokine exposure	-Apoptosis, -Necrosis, -Viability, ↓ IL-1β, ↓ MCP-1, ↓ IL-15, ↓ IP-10, ↑ VDR, ↑ Calbindin
Wolden-Kirk et al. [59]	Rat β-cell line (INS-1E)	1,25(OH)_2_D at 10 nM before or concurrently with cytokine exposure for 24 h	-Apoptosis
Wei et al. [60]	hiPSC-derived β-like cells Rat β-cell line (INS-1)	shRNA KO of VDR	↑ Apoptosis, ↓ NKX6-1, ↓ Ins2, ↓ PDX1
*H*_*2*_ *O*_*2*_ */cadmium-induced oxidative stress*			
Deng et al. [63]	Mouse β-cell line (NIT-1)	1,25(OH)₂D at 1 and 10 nM concurrently with cadmium exposure	↑ Viability, ↑ Insulin, ↑ IRS-1 & − 2, ↓ ROS
Chen et al. [64]	Human β-cell line (EndoC-βH1)	1,25(OH)₂D at 100 nM for 24 h VDR siRNA, VDR overexpression, & H305Q mutant VDR	VDR siRNA/mutant VDR: ↓ Viability, ↑ FoxO1 VDR overexpression/vit D treatment: ↓ Apoptosis, ↓ FoxO1, ↑ Viability
Hu et al. [65]	Mouse β-cell line (MIN6)	1,25(OH)₂D at 5 nM for 3 h before H₂O₂ exposure	↓ Apoptosis, ↑ Viability, ↓ p-PERK, ↓ Caspase-3 & -9, ↓ Bax, ↑ Bcl-2
*STZ-induced cytotoxicity*			
He et al. [66]	Rat β-cell line (INS-1)	1,25(OH)_2_D at 100 nM for 24 h	↑ Viability, ↑ Autophagy, ↓ ROS, ↑ LC3-II, ↑ Beclin-1
Zheng et al. [67]	Mouse β-cell line (MIN6)	1,25(OH)_2_D at 0.5 nM for 24 h	↓ Pyroptosis, ↑ Proliferation, ↑ Insulin, ↓ Caspase-1, ↓ NLRP3, ↓ NF-κB, ↓ IL-1β, ↓ IL-18
*Glucotoxic stress*			
Bornstedt et al. [68]	Rat β-cell line (INS-1E)	1,25(OH)_2_D/25(OH)D at 10 nM for 72 h before glucose exposure (22 mM)	↑ Serpina3, ↑ Bmp3, ↑ Igf1, ↓ Casp3, ↓ Cxcl10, ↓ Foxo3
Wu et al. [72]	Rat β-cell line (INS-1E)	1,25(OH)_2_D treatment concurrently with glucose exposure (30 mM)	↓ Pyroptosis, ↓ Bax:Bcl-2, ↓ Caspase-1 & -8, ↓ ROS, ↓ NLRP3, ↓ IL-1β, ↓ TXNIP, ↓ mTOR ↑ AMPK
Zhao et al. [73]	Rat β-cell line (INS-1)	1,25(OH)_2_D at 100 nM with glucose exposure (33 mM) for 48 h	↓ Apoptosis, ↓ FTH, ↓ NF-κB, ↓ DMT1
Ding & Wu [74]	Rat β-cell line (INS-1)	1,25(OH)_2_D at 100 nM after glucose exposure (30 mM) for 24 h VDR siRNA	↓ Apoptosis, ↑ Viability, ↓ ROS, ↓ ACSL4, ↓ FOXO1, ↑ VDR, ↑ GPX4
Lipotoxic stress			
Zhang et al. [75]	Mouse β-cell line (MIN6)	1,25(OH)_2_D at 1, 2, 4 nM before palmitic acid exposure (0.5 mM)	↓ Apoptosis, ↓ ROS, ↓ ER stress, ↓ TNF-α, ↓ IL-6, ↓ MDA, ↑ SOD, ↑ GSH

↓ decreased, ↑ increased, and-indicates-no change

*ACSL4* acyl-CoA synthetase long-chain family member 4, *AMPK* AMP-activated protein kinase, *DMT1* divalent metal transporter 1, *GSH* glutathione, *MAPK* mitogen-activated protein kinase, *MDA* malondialdehyde, *mTOR* mammalian target of rapamycin, *NO* nitric oxide, *ROS* reactive oxygen species, *SOD* superoxide dismutase, *STZ* streptozotocin, *VDR* vitamin D receptor

Table 2 summarises islet and ex-vivo experiments. In a single study by Galbiati et al., 1,25(OH)_2_D suppressed insulin expression and secretion via rapid, non-genomic ERK1/2 phosphorylation without inducing apoptosis, contrasting with its pro-apoptotic action in βTC insulinoma models. Under cytokine-induced inflammatory stress, Vitamin D signalling acts as a survival factor in human and mouse islets, but not in rat islets. This anti-apoptotic protection is driven by the up-regulation of A20, inhibition of IKKα/β and NF-κB pathways, and suppression of the iNOS/NO axis and Fas receptor.

**Table 2:** Islet and ex-vivo studies of vitamin D signalling on pancreatic β-cell apoptosis and survival

Study	Islet/ex-vivo model	Treatments	Outcomes
*Human insulinoma cells and islets*			
Galbiati et al. [53]	Primary human benign insulinoma cells Isolated human islets	1,25(OH)₂D at 10, 100, & 1000 nM for 48 and 72 h	Insulinoma: -Apoptosis, ↓ Insulin Human islets: -Apoptosis, ↓ Insulin, ↑ p-ERK1/2
*Cytokine-induced inflammatory stress*			
Riachy et al. [56]	Isolated human islets	1,25(OH)₂D 10 or 1000 nM with or before cytokine exposure for 48 h	↓ Apoptosis, ↓ IKKα/β, ↑ A20
Gysemans et al. [58]	Isolated rat pancreatic β-cells	1,25(OH)₂D at 10 nM for 24 h before cytokine exposure	-Apoptosis, -Necrosis, -Viability
Riachy et al. [57]	Isolated human islets	1,25(OH)₂D at 10 & 1000 nM concurrently with cytokine exposure for 48 h	↓ Apoptosis, ↓ NO, ↓ NF-κB, ↓ Caspases, ↓ Fas, ↓ SMAC, ↑ API3, ↑ DSPI/GILZ
Wolden-Kirk et al. [59]	Isolated human islets Isolated mouse pancreatic islets	Human islets: 1,25(OH)₂D at 10 nM pre-cytokine exposure for 24 h Mouse islets: 1,25(OH)₂D at 10 nM with cytokine exposure for 6 or 24 h	↓ Apoptosis, ↓ NF-κB, ↓Chemokines, ↓ Cytokines, ↑ GSIS
Wei et al. [60]	Isolated mouse pancreatic islets	1,25(OH)₂D analogue calcipotriol (Cal)	↓ Inflammatory response, ↓ Apoptotic signalling pathway, ↑ Insulin

↓ decreased, ↑ increased, and -indicates no change

*API3* apoptosis inhibitor 3, *DSPI* desmoplakin I, *GILZ* glucocorticoid-induced leucine zipper, *GSIS* glucose-stimulated insulin secretion, *SMAC* second mitochondria-derived activator of caspases, *NO* nitric oxide

### Animal studies, in vivo

#### RIP1Tag2 insulinoma model

Using the RIP1Tag2 mouse model of pancreatic β-cell carcinogenesis, Galbiati and colleagues showed in vivo antitumorigenic activity of calcitriol. They found that calcitriol induced a three-fold increase in the rate of apoptosis within the tumours following three weeks of treatment, while the rate of cell proliferation remained unchanged [53].

#### Non-obese diabetic mice

Gysemans et al. found that the NOD mouse model of T1D treated with 1,25(OH)₂D had significantly reduced insulitis compared to controls, with reduced mean insulitis score at 10 weeks from 2.06 to 0.55. This protection was attributed to the reduced intra-islet expression of *Il1b*, *Cxcl10* (IP-10), and *Il15*. However, 1,25(OH)₂D failed to protect the isolated islets of NOD-SCID mice (which lack T, B, and NK cells) from direct cytokine-induced cell death, suggesting that the effect in NOD mice may be immune cell mediated [58].

In the Ins2-/- NOD mouse model, which features an accelerated and aggressive form of T1D, early treatment with the vitamin D analogue 2α-methyl-19-nor-(20S)− 1α,25-dihydroxyvitamin D3 (2AMD) at 3 weeks of age significantly prevents disease onset. By 21 weeks, all vehicle-treated mice had developed diabetes, whereas only 37.5% of 2AMD-treated mice were diabetic. This intervention works by limiting intra-islet invasion of CD4+ and CD8+ T cells, thereby preserving islet integrity and maintaining normal blood glucose levels [76].

Likewise, in another NOD mice study, Vitamin D supplementation induced an anti-apoptotic effect on β-cells by upregulating the survival protein Bcl-2 while decreasing pro-apoptotic markers like Bax and cleaved caspases-3 and − 9. Vitamin D also reduced pancreatic IL-2, TNF-α, and IFN-γ levels, while IL-10 was largely unchanged. Most notably, Vitamin D specifically downregulates the expression of Cathepsin G (CatG) which inhibits CD4+ T cell activity and preserves β-cell function [77].

#### STZ-induced diabetic stress

STZ is a chemical compound that is taken up by and destroys β-cells which is widely used to induce diabetes in mice and rats [78]. Wang and researchers reported that 1,25(OH)₂D reduced both diabetes incidence and degree of hyperglycemia in STZ-injected mice by decreasing the inflammatory infiltration of islets and attenuating the decline in plasma insulin levels [79].

Similarly, in an STZ-induced diabetic rat model, 1,25(OH)₂D partially preserved β-cell mass and function. β-cell density increased from 4.03 ± 0.76 cells/mm^2^ in STZ-treated rats to 6.10 ± 1.36 cells/mm^2^ following 1,25(OH)₂D₃ treatment, representing an approximately 51% increase. Although apoptosis was not directly measured, 1,25(OH)₂D₃ reduced pancreatic TNF-α and *Bax* expression and lowered the *Bax/Bcl-2* ratio [80].

Using various diabetic models including db/db B6, db/db BKS, Ins2-IAPP transgenic, and STZ-induced mice, Wei et al. reported that significant therapeutic benefits were achieved through the combination of iBRD9 and Cal [60]. In db/db BKS mice, 12 weeks of Cal+iBRD9 treatment increased pancreatic islet area by approximately 70%, doubled mean islet size. This dual-action approach restored β-cell identity, promoted islet proliferation, and reduced progressive cell death. These findings highlight the critical role of chromatin remodelling via iBRD9 in ensuring the genomic accessibility required for the VDR to reach and activate its target genes [81].

β-cell-specific VDR overexpression in Tg3 transgenic mice resulted in a lower incidence of STZ-induced diabetes, while increased VDR levels did not prevent direct STZ-induced apoptosis. Instead, they significantly preserved β-cell mass and elevated insulin levels by enhancing cellular proliferation and attenuating inflammation, evidenced by an approximately threefold increase in Ki67-positive β-cells, increased *A20* expression, and reduced expression of *Tnfa*, *Tgfb*, and *H2-Aa* [82].

The overexpression of the E3 ubiquitin ligases RNF20 or RNF40 partially restored pancreatic VDR expression and significantly ameliorated hyperglycemia, hypoinsulinaemia, and systemic inflammation. This regulatory axis also suppressed STZ-induced apoptosis and reduced expression of the pro-apoptotic proteins Bax, cleaved caspase−3, and cleaved PARP, which was completely abolished in VDR knockout models, confirming VDR-dependent signalling actions [83].

#### High-fat diet (HFD) & STZ-induced diabetic stress

Many researchers use a combination of HFD and STZ, often at lower dose, to replicate the metabolic complexities of T2D [84]. In this model, cholecalciferol improved islet morphology and reversed cellular hallmarks of the disease, such as β-cell destruction and amyloidosis-like deposits. Mechanistically, cholecalciferol was associated with a less pro-apoptotic islet profile, as indicated by a reduction in cleaved caspase−3 immunoreactivity. Vitamin D also reduced pancreatic IL-6 and MDA, while increasing GSH, SOD, catalase, and total antioxidant capacity [85].

Beyond correcting lipid profiles and ameliorating metabolic dysfunction, 1,25(OH)₂D restored insulin-positive β-cell staining and reduced TUNEL-defined cell death in pancreatic tissue. These benefits were accompanied by lower ROS and MDA levels, and increased endogenous antioxidant activity [75].

Jia and colleagues reported a synergistic effect of vitamin D3 and glibenclamide in managing T2D in a rat model, significantly reducing blood glucose levels and enhancing insulin secretion beyond the capabilities of monotherapy. The combination markedly reduces the rate of islet-cell apoptosis, associated with lower NF-κB p65 immunoreactivity and reduced pancreatic *Fas*, *Tnf-α*, *Il1b*, and *Ifng* mRNA expression [86].

In addition, vitamin D increased CD49a-positive β-cell marker staining and reduced oxidative and ferroptosis-associated changes, including elevated ROS and iron accumulation. These changes were accompanied by favourable regulation of GPX4 and ACSL4 [74].

Furthermore, in early stages of diabetes, Vitamin D3 improved glucose homeostasis and preserved islet morphology. This protection is achieved by inhibiting STZ-induced NF-κB signalling and, crucially, suppressing the GSDMD pathway. By blocking GSDMD-mediated pyroptosis, vitamin D significantly reduced the local inflammatory response, a protective effect that mirrors the results seen in GSDMD-knockout models [67].

#### Other models

In rats fed a high-glucose diet (HGD), supplementation with vitamin D3 significantly lowers blood glucose levels and enhances glucose tolerance compared to untreated HGD controls. Vitamin D3 prevents the islet atrophy and the loss of insulin-producing cells typically triggered by chronic high-glucose stress [72].

Likewise, in 1,25(OH)₂D facilitated significant β-cell mass preservation in Zucker Diabetic Fatty rats, evidenced by restored islet morphology and increased insulin-positive signalling. Consistent with their in vitro results, and the above paper about ferroptosis, the central mechanism in that recovery was the reduction of pancreatic iron deposition [73].

## Summary of animal studies

In vivo studies investigated the therapeutic potential of Vitamin D are shown in Table 3. In the RIP1Tag2 mouse model of β-cell carcinogenesis, calcitriol treatment exerts antitumorigenic activity by driving in intratumor apoptosis. In NOD mouse models of autoimmune diabetes, Vitamin D and its analogues provided robust protection in islet integrity by disrupting chemoattractive signals and preventing intra-islet T-cell invasion. Vitamin D supplementation also induced an anti-apoptotic and anti-inflammatory environment within the pancreas of these mice. In STZ-induced diabetic rodents, 1,25(OH)₂D reduced disease incidence, ameliorated hyperglycemia, and preserved functional β-cell mass. Genetic models further proved these VDR-dependent actions; transgenic over-expression of VDR (Tg3 mice) or its indirect upregulation via the RNF20/RNF40 axis protected animals from STZ-induced diabetes by shifting the cellular balance toward proliferation and suppressing systemic inflammation. Crucially, in aggressive diabetic models (such as db/db and Ins2-IAPP transgenic mice), maximising the therapeutic efficacy of Vitamin D requires co-treatment with an iBRD9 inhibitor, which drives chromatin remodeling to ensure genomic accessibility for the VDR.

**Table 3:** In vivo studies of vitamin D signalling on pancreatic β-cell apoptosis and survival

Study	Animal Model (Mice unless specified)	Treatments	Outcomes
RIP1Tag2 Insulinoma Model			
Galbiati et al. [53]	RIP1Tag2 transgenic	1,25(OH)_2_D at 5 µg/kg 2nd daily for 3 weeks	↑ Apoptosis, - Proliferation, ↓ Insulin
NOD mice			
Gysemans et al. [58]	NOD NOD-SCID	1,25(OH)_2_D at 5 µg/kg every 2 days for 10w	NOD mice: ↓ Insulitis, ↓ *Il1b*, ↓ *Il15*, ↓ MCP-1 NOD-SCID: - Apoptosis, - Necrosis, - Viability, ↓ *Il15*, ↓ IP-10
Kiekhaefer et al. [76]	Ins2−/− NOD	2α-methyl-19-nor-(20S)-1α,25-dihydroxyvitamin D3 (2AMD) at 600 pg/d	↑ Islet integrity, ↑ Insulin, ↓ T-cell infiltration
Lai et al. [77]	NOD	Feed supplemented with vitamin D at 2,200 IU/kg for 28 days	↓ Apoptosis, ↓ Caspase−3 & − 9, ↓ Bax, ↓ TNF-α, ↓ IL-2, ↓ IFN-γ, ↓ CatG, ↑ Bcl-2, ↑ Insulin
STZ-induced diabetic stress			
Wang et al. [79]	C57BL/6	1,25(OH)₂D 5 µg/kg every 2nd day	↓ β-cell destruction, ↓ Inflammation, ↑ Body weight, ↑ Insulin
Su et al. [80]	SD rats	1,25(OH)₂D 1 µg/kg/d for 4 weeks	↓ β-cell destruction, ↑ GSIS, ↓ TNF-α, ↓ *Bax/Bcl-2*
Wei et al. [60]	B6 db/db BKS db/db C57BL/6 J Ins2-IAPP transgenic	Cal at 60 µg/kg & iBRD9 at 10 mg/kg 3 times per week for 9 weeks	db/db B6: ↓ BGL, ↑ Insulin, ↑ Nkx6-1, ↑ Nr4a1 db/db BKS: ↓ BGL, ↑ Insulin, ↑ Islet proliferation, ↓ ER stress STZ mice: ↓ β cell death, ↑ Insulin, ↑ NKX6-1 h-IAPP mice islets: ↓ Casp1, ↓ Ccl2, ↓ Cxcl1, ↓ Il1b
Morró et al. [82]	Tg3 transgenic	VDR overexpression	— Apoptosis, ↑ proliferation, ↑ Insulin, ↑ *A20*, ↓ Inflammation, ↓ *Tnfa*, ↓ *Tgfb*, ↓ *H2-Aa*
Wu et al. [83]	C57BL/6	RNF20/RNF40 overexpression → VDR upregulation	↓ Apoptosis, ↓ Bax, ↓ Caspase−3, ↓ PARP, ↓ BGL, ↑ Insulin, ↓ Inflammation
HFD & STZ-induced diabetic stress			
Fathi et al. [85]	Wistar albino rats	Cholecalciferol 10 IU/kg/d for 3d after T2D induction	↓ β-cell destruction, ↓ IL-6, ↓ Cortisol, ↓ Caspase−3, ↓ eNOS, ↓ MDA, ↑ Antioxidants (SOD, CAT, GSH), ↑ Insulin
Ding and Wu [74]	SD rats	1,25(OH)_2_D at 0.03 µg/kg/d for 4 weeks	↑ β-cell mass, ↓ Ferroptosis, ↓ ROS, ↓ ACSL4, ↑ GPX4
Zhang et al. [75]	C57BL/6	1,25(OH)_2_D at 150, 300, & 600 ng/kg/d for 12w	↓ Apoptosis, ↓ BGL, ↓ ROS, ↓ MDA, ↑ SOD, ↑ GSH
Jia et al. [86]	SD rats	D3 500 IU/kg/day + glibenclamide for 8w	↓ Apoptosis, ↓ BGL, ↑ Insulin, ↓ p65, ↓ *Tnf-α*, ↓ *Il1b*, ↓ *Ifng*, ↓ *Fas*
Zheng et al. [67]	GSDMD-/- C57BL/6 J	1,25(OH)₂D at 5 µg/kg every other day for 3w	↓ Pyroptosis, ↓ Caspase-1, ↓ NLRP3, ↓ NF-κB, ↓ IL-1β, ↓ IL-18, ↓ BGL, ↓ HbA1c
Other models			
Wu et al. [72]	SD rats	Cholecalciferol at 233.3 U/kg per week	↓ Islet atrophy, ↓ Hyperglycemia, ↑ IPGTT, ↑ GSIS
Zhao et al. [73]	ZDF rats	1,25(OH)₂D at 8 µg/kg 2nd daily for 7w	↑ β-cell mass, ↓ Caspase−3, ↓ NF-κB, ↓ FTH & FTL, ↓ DMT1, ↓ ZIP14

↓ decreased, ↑ increased, and -indicates no change

*ACSL4* acyl-CoA synthetase long-chain family member 4, *BGL* blood glucose level, *DMT1* divalent metal transporter 1, *ER* endoplasmic reticulum, *FTH* ferritin heavy chain, *FTL* ferritin light chain, *GPX4* glutathione peroxidase 4, *GSDMD* gasdermin D, *GSH* glutathione, *GSIS* glucose-stimulated insulin secretion, *HFD* high-fat diet, *IPGTT* intraperitoneal glucose tolerance test, *MDA* malondialdehyde, *NOD* non-obese diabetic, *ROS* reactive oxygen species, *SOD* superoxide dismutase, *STZ* streptozotocin, *VDR* vitamin D receptor, *ZDF* Zucker diabetic fatty

In rodent models combining a HFD and STZ to replicate the metabolic complexities of T2D, Vitamin D treatment consistently preserves pancreatic histopathology, restores islet morphology, and reverses severe cellular hallmarks such as pancreatic destruction and amyloidosis-like deposits. Whether administered as cholecalciferol or 1,25(OH)₂D, it exerts robust anti-inflammatory, anti-apoptotic, and antioxidant effects that suppress β-cell death, correct lipid profiles, and mitigate ER stress. Consistently in HGD-fed rats and Zucker Diabetic Fatty rats, Vitamin D supplementation enhanced glucose tolerance prevented islet degradation, and facilitated significant functional β-cell mass recovery.

## Discussion

Research into the extra-skeletal effects of Vitamin D, particularly regarding its modulation of essential cellular processes, has expanded exponentially over the past few decades [6, 8, 87, 88]. To our knowledge, this is the first systematic review to comprehensively synthesize the literature investigating the role of vitamin D signalling in pancreatic β-cell apoptosis. Of the 26 studies analysed, substantial heterogeneity was found across experimental models. The majority of the literature evaluated models utilising a variety of in vitro, ex vivo, and in vivo stressors to replicate the pathological conditions of T1D and T2D.

Responses to vitamin D signalling varied between β-cell models. Increased apoptosis was reported specifically in βTC insulinoma models, whereas other insulinoma-derived cell lines, including RINm5F, MIN6, and INS-1/INS-1E, showed neutral or cytoprotective responses under inflammatory, oxidative, or metabolic stress. In primary islets and in vivo models, most studies reported reduction in β-cell apoptosis or cell death of β-cells under various cytotoxic challenges and rodent diabetic models.

### Vitamin D’s anti-inflammatory effect in β-cells

Inflammation and apoptosis are intricately connected and can set up a feed-forward loop that promotes diabetes [89, 90]. NF-κB is a primary inducer of cytokine-induced β-cell death, as its inactivation reduces toxic iNOS transcription [91], whereas its activation by cytokine combinations of LIGHT and IFN-γ triggers a caspase-dependent mitochondrial apoptotic pathway [92].

Wei et al. provide a mechanistic framework for the anti-inflammatory effect of vitamin D in pancreatic β-cells [60]. They demonstrated that VDR protects β-cells by switching its association from a BRD9-containing BAF complex to a BRD7-containing PBAF complex. This switch increases chromatin accessibility at VDR-responsive loci and promotes an anti-inflammatory transcriptional programme, including the induction of Nfkbia, which suppresses NF-κB-driven cytokine damage [60].

Indeed, the primary molecular gatekeeper of Vitamin D's anti-inflammatory action across most models is the suppression of the NF-κB pathway. Vitamin D treatment enhances the expression of A20/TNFAIP3 [56, 82], an anti-inflammatory regulator that disrupts TNFR1 signalling through various mechanisms [93]. It binds to M1-linked polyubiquitin chains, preventing the activation of downstream cascades by outcompeting other ubiquitin-binding proteins [94, 95]. A20 also functions as a deubiquitinating enzyme, actively removing K63-linked polyubiquitin chains from key signalling factors such as RIPK1 and NEMO, thereby halting the cascade necessary for activating the IKK complex [96–98]. Moreover, A20 opposes the deubiquitinase CYLD from degrading M1-linked ubiquitin chains, stabilizing TNF-TNFR1 recruited Complex I members and preventing its conversion into cytosolic death complexes (Complex IIa/IIb) that initiate caspase−8-mediated apoptosis [93].

β-cells are highly susceptible to the accumulation of misfolded proteins within the ER lumen, known as ER stress, which triggers the unfolded protein response (UPR) and activates inflammatory networks [99, 100]. When chronically sustained, the UPR transitions from an adaptive survival mechanism to a pro-apoptotic cascade [101]. The literature demonstrates that Vitamin D mitigates this structural degradation by downregulating ER stress markers such as GRP78 and CHOP [60, 65, 75]. This stabilisation of ER homeostasis occurs concurrently with reduction in the local secretion of inflammatory cytokines including TNF-α, IL-2, IL-6, IL-1β, IFN-γ, and IL-18 [58, 67, 72, 75, 77, 80, 85, 86]. In addition, Vitamin D also operates at a systemic level by suppressing the expression of chemo-attractive signals and CatG, preventing the infiltration of inflammatory T-cells [75, 77]. Therefore, in metabolically or inflammatory stressed β-cells, vitamin D may reduce apoptosis indirectly by resolving the upstream inflammatory and ER-stress signals that drive cell death.

### Heterogeneity of vitamin D’s effects on β-cell survival

**Fig. 3:**
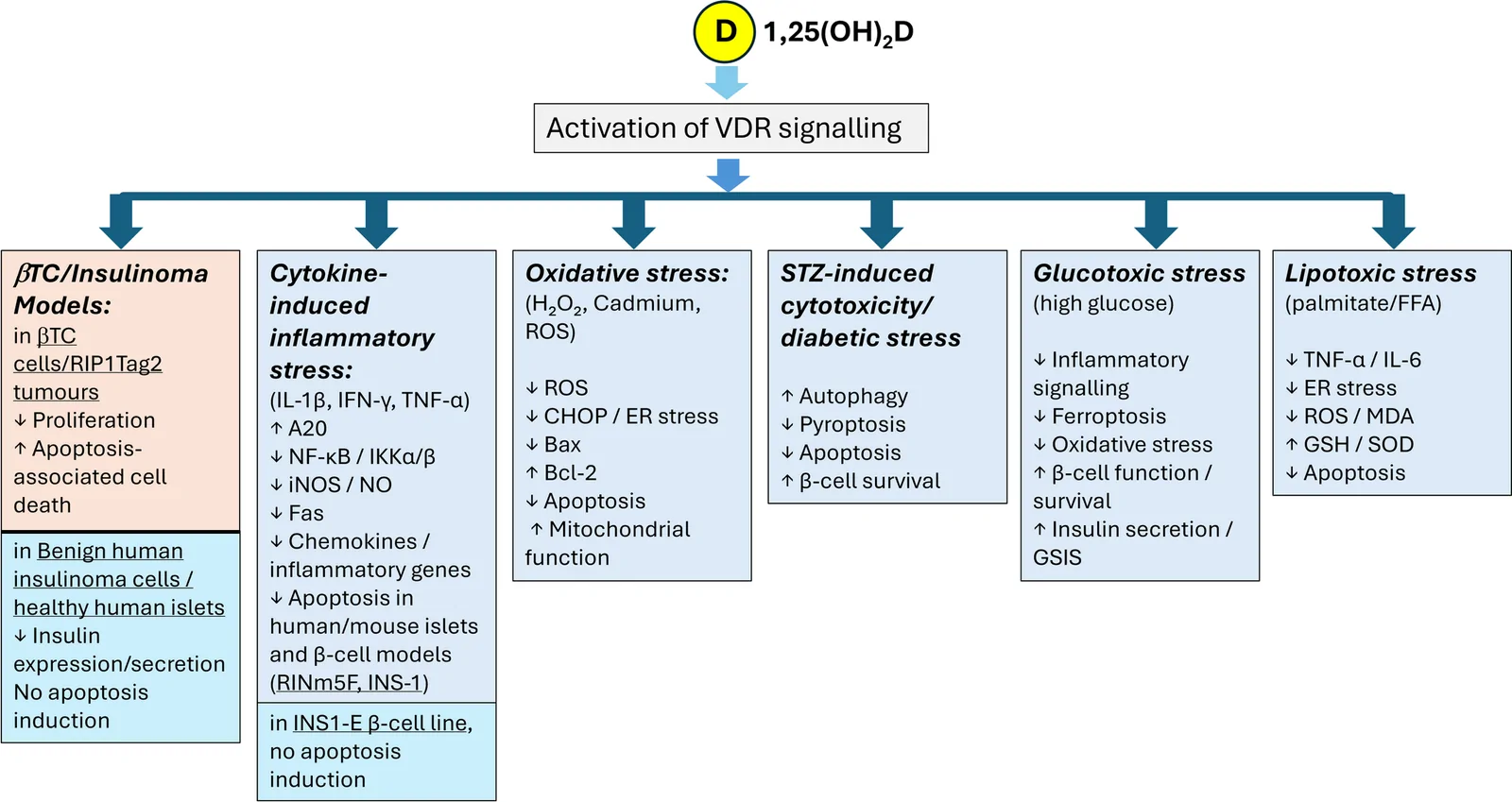
Heterogeneous, context-dependent effects: The effects of 1,25(OH)_2_D/VDR on b-cells vary with cell type, stressor, and experimental conditions, including species, dose, and timing Vitamin D/VDR signalling elicits heterogeneous β-cell responses that vary with cellular context, stress conditions, and treatment parameters. Reported outcomes range from pro-survival effects to limited/no response or pro-death/anti-proliferative effects, indicating that vitamin D/VDR effects are not universal across experimental β-cell models. KD, knockdown; OE, overexpression; PA, palmitic acid; STZ, streptozotocin; VDR, vitamin D receptor.

The regulatory effects of Vitamin D on programmed cell death are profoundly context- and tissue-dependent. Out of the 26 studies included in this review, Galbiati and researchers found that calcitriol increased apoptosis-associated cell death and inhibited proliferation in βTC insulinoma cells and increased apoptosis in RIP1Tag2 β-cell tumours [53, 54]. However, this effect should not be interpreted as a simple distinction between tumorigenic and non-tumorigenic β-cells. Notably, the same group reported that calcitriol did not induce apoptosis in primary benign human insulinoma cells. Several other models included in this review, including RINm5F, INS-1/INS-1E, NIT-1 and MIN6 cells, are also insulinoma-derived, yet displayed neutral or cytoprotective responses to vitamin D under inflammatory, oxidative or metabolic stress [56–60, 63, 64, 67, 72–77, 79, 80, 82, 83, 85, 86]. The reasons for these divergent findings remain uncertain. Experimental differences include model background, the presence of cytotoxic stressors, vitamin D concentration, duration of the treatment, and assays used to define cell death (Fig. 3).

Context-dependent effects of vitamin D on apoptosis have also been reported in other tissues and conditions, although these observations do not directly explain the divergent responses observed among the β-cell models included in this review. As previously mentioned, in osteoarthritis models, vitamin D suppresses chondrocyte apoptosis and cartilage degradation by activating AMPK/mTOR-driven autophagy and inhibiting NF-κB nuclear translocation [29, 102, 103]. However, in the highly proliferative environments of rheumatoid arthritis (RA), vitamin D reverses this protective trend to actively promote apoptosis; it inhibits hyperplastic NF-κB survival signalling in RA chondrocytes and enhances p53 acetylation by inhibiting Sirt1 in fibroblast-like synoviocytes, thus restoring executioner caspase activity to counteract destructive joint hyperplasia [32, 33].

The heterogeneous effects of vitamin D signalling on apoptosis may initially appear contradictory. In normal signalling, 1,25(OH)₂D binds to VDR, which then interacts with retinoid X receptor (RXR) to regulate gene expression through vitamin-D-responsive genomic regions [104]. However, VDR's activity is not independent; its output is influenced by the cell's identity, chromatin state, the presence of co-activators and co-repressors, inflammatory transcription factors, and signalling environment [105, 106]. For instance, genome-wide analysis revealed that ligand-bound VDR binds thousands of genomic sites, but the VDR cistrome is highly cell-specific, with only a small subset of sites shared across cell types [107]. The expression of vitamin D-metabolising enzymes, CYP27B1 and CYP24A1, differs across tissues and pathological states, leading to same circulating vitamin D levels with different degrees of local VDR activation [108, 109]. In normal β-cells, where apoptosis is primarily driven by pathological stress—such as cytokines or metabolic overload, as discussed in this review—vitamin D serves as a protector by suppressing inflammatory mediators and inflammasome-related signalling [56–60, 67, 72–74, 76, 77, 79, 80, 82, 83, 85, 86], mitigating ER-stress pathways [60, 75, 83], reducing ROS [63, 64, 72, 75, 83, 85], and preserving β-cell identity [60, 63]. At the mitochondria level, vitamin D could shift the balance away from pro-apoptotic signalling to anti-apoptotic survival proteins [57, 64, 72, 77, 80, 83].

Differences in experimental conditions may contribute to the heterogeneous responses observed across β-cell models. Although βTC, MIN6, and INS-1/INS-1E cells are all immortalised and insulinoma-derived cell lines with proliferative characteristics, the presence of inflammatory, oxidative, glucotoxic, or lipotoxic stress may substantially alter the transcriptional and signalling state of β-cells. Human islets and EndoC-βH1 cells exposed to IL-1β and IFN-γ displayed extensive remodelling of the β-cell regulatory landscape, including cytokine-responsive enhancers, altered chromatin accessibility and changes in 3D chromatin structure [110]. Prolonged exposure to high glucose in βTC-6 cells could suppress IRS-2/PI3K signalling and impair Akt activation [111]. Likewise, palmitate-induced ER stress inhibited Akt signalling through tribbles 3 (TRB3) and FoxO3a activation [112]. These stress-induced changes may modify the transcription factors, cofactors, and regulatory regions available to VDR and thereby influence the cellular consequences of VDR activation. VDR transcriptional activity itself is context-dependent and has been shown to regulate cell-cycle genes such as p21 and p27 differently in cancer cells [113]. Additionally, Campbell et al. reported that mutant p53 is capable to interact with VDR and modulate VDR-regulated genes [114]. Thus, differences in the cellular stress environment may contribute to the contrasting responses to vitamin D observed between the βTC/RIP1Tag2 models by Galbiati et al. and other insulinoma-derived β-cell lines. However, whether these stress-induced mechanisms directly modify VDR activity and are responsible for the divergent responses have not been experimentally established.

### Relevance and evidence from human studies

In this review, only three studies evaluated the effects of vitamin D in primary human islets exposed to cytokine-induced inflammatory stress [56, 57, 59]. For obvious reasons, longitudinal biopsy sampling of the human pancreas is not feasible. Thus, there is no direct evidence that vitamin D supplementation reduces β-cell apoptosis or preserves β-cell mass in humans. Nevertheless, clinical and epidemiological studies provide biological support for an association between vitamin D status and diabetes risk or β-cell function.

Vitamin D deficiency, particularly during early life, is associated with an increased risk of T1D [115]. The EURODIAB multicentre case–control study included 820 children with T1D and 2335 controls and found that vitamin D supplementation during infancy was associated with a lower risk of disease [116]. In the Northern Finland 1966 birth cohort, 10,366 children were followed from infancy to 31 years of age. Regular vitamin D supplementation (2,000 IU/day) during the first year of life was associated with a markedly lower risk of T1D than no supplementation [117]. Likewise, the TEDDY prospective cohort concluded that higher childhood serum 25(OH)D levels were associated with lower islet-autoimmunity risk [118]. In a participant-blinded, placebo-controlled trial, supplementation of active vitamin D analogue alfacalcidol (0.50 μg/day) was associated with higher fasting C-peptide concentrations and lower insulin requirements in children aged 8–15 years with newly diagnosed T1D [119]. However, the IMDIAB XIII double-blind, placebo-controlled trial, involving 34 individuals aged 11–35 years with recent-onset T1D, found no beneficial effect of calcitriol supplementation (0.25 μg/day) on preservation of residual β-cell function [120].

Consistently, people with T2D have a higher prevalence of vitamin D deficiency [48]. The CaDDM randomised controlled trial concluded that vitamin D supplementation (2,000 IU/day) improved β-cell function in adults at high risk of T2D [121]. Similarly, another double-blind randomised trial involving 96 adults with newly diagnosed T2D showed improved peripheral insulin sensitivity and β-cell function after six months of vitamin D3 supplementation at 5,000 IU/day [122]. In contrast, cholecalciferol supplementation (20,000 IU/week) did not slow down diabetes progression in a large scale randomised controlled trial of a largely vitamin D-sufficient participants [123]. Likewise, 4,000 IU/day of vitamin D3 supplementation did not significantly reduce progression to T2D in a randomised controlled trial involving 2,423 adults with prediabetes [124].

Collectively, the human evidence does not directly confirm the anti-apoptotic actions of vitamin D identified in experimental β-cell models. One possible explanation is that vitamin D acts primarily by limiting ongoing β-cell apoptosis and related inflammatory, oxidative, or metabolic stress, rather than by regenerating β-cells that have already been lost. Therefore, the magnitude of benefit may depend on disease stage and residual β-cell reserve. Supplementation initiated after substantial or irreversible β-cell loss is unlikely to restore β-cell mass, which may explain the inconsistent results of human supplementation studies.

### Limitations

This review has several limitations. First, the evidence is predominantly preclinical, with most studies using immortalised β-cell lines, and fewer using isolated islets, or in vivo rodent models. As mentioned previously, human pancreatic biopsy studies are not available. In studies using primary human islets, donor vitamin D status, geographical location, season of tissue collection, and sunlight exposure were not consistently reported, which may represent potential sources of biological heterogeneity. Second, the pro-apoptotic role of vitamin D in insulinoma β-cell models is supported by relatively few studies, though it is of interest in the context of the generally increasing rates of pancreatic cancer and vitamin D deficiency. Third, the interventions were highly variable, including cholecalciferol, calcitriol, vitamin D analogues, VDR overexpression, VDR knockdown, and combination strategies such as calcipotriol with iBRD9. Dosage, treatment timing, and exposure duration also differed across studies, limiting the ability to define a therapeutic dosage. Finally, studies used different markers, including caspase activity, TUNEL staining, histological analysis, DNA fragmentation, BAX/BCL-2 ratio, and cell viability assays, which may capture overlapping but distinct forms of cell injury. This is particularly relevant as several studies also implicated pyroptosis, ferroptosis, and autophagy-related survival pathways rather than classical apoptosis alone.

## Conclusion

This systematic review highlights vitamin D signalling as a regulator of pancreatic β-cell survival and apoptosis. Across models of β-cell stress, including cytokine-induced inflammation, oxidative stress, glucotoxicity, lipotoxicity, STZ-induced injury, and diabetic rodent models, vitamin D or VDR activation generally reduced β-cell apoptosis and preserved β-cell function. However, divergent responses were observed in βTC insulinoma models, which may reflect variation in cellular background, vitamin D treatment, and experimental stressors. Further studies using matched experimental conditions are required to define the context-dependent effects of vitamin D signalling on β-cell survival.

## Supplementary Information

Below is the link to the electronic supplementary material.

Supplementary Material 1

## Data Availability

No datasets were generated or analysed during the current study.
