# Supplementary material for: The role of vitamin D signalling in β cell apoptosis: a systematic review
Source: Apoptosis. 2026 Oct 1;31(10):237. doi: 10.1007/s10495-026-02453-5 (PMC13630944; doi:10.1007/s10495-026-02453-5)
Supplement: Supplementary file 1 [file 10495_2026_2453_MOESM1_ESM.docx]

Supplementary Table 1. Search strategy 12 Dec 2025

| **Data Base** | **Results** |
| --- | --- |
| **Medline** |  |
| Vitamin D/ OR (vitamin d or vitamin d receptor or cholecalciferol or calcitriol).mp. AND Apoptosis/ OR (apoptosis or programmed cell death or cell death).mp. AND Insulin-Secreting Cells/ or "Islets of Langerhans"/ OR (beta cell* or islet cell* or pancreatic beta cell*).mp. | 157 |
| **Embase** |  |
| vitamin D/ OR (vitamin d or vitamin d receptor or cholecalciferol or calcitriol or ergocalciferol).ti,ab. AND pancreas islet beta cell/ OR (beta cell* or islet cell* or pancreatic beta cell*).ti,ab. AND apoptosis/ OR (apoptosis or programmed cell death or cell death).ti,ab. | 83 |
| **Scopus** |  |
| (“vitamin d” OR “vitamin d receptor” OR cholecalciferol OR calcitriol) AND (“beta cell*” OR “islet cell*” OR “pancreatic beta cell*”) AND (apoptosis OR “programmed cell death” OR “cell death”) | 99 |

Supplementary Table 2. PRISMA Checklist

| **Section and Topic** | **Item #** | **Checklist item** | **Location where item is reported** |
| --- | --- | --- | --- |
| **TITLE** | | |  |
| Title | 1 | Identify the report as a systematic review. | Title page |
| **ABSTRACT** | | |  |
| Abstract | 2 | See the PRISMA 2020 for Abstracts checklist. | Abstract page |
| **INTRODUCTION** | | |  |
| Rationale | 3 | Describe the rationale for the review in the context of existing knowledge. | Introduction, paragraph 3-4 |
| Objectives | 4 | Provide an explicit statement of the objective(s) or question(s) the review addresses. | Introduction, paragraph 5 |
| **METHODS** | | |  |
| Eligibility criteria | 5 | Specify the inclusion and exclusion criteria for the review and how studies were grouped for the syntheses. | Method, 2.1 Eligibility criteria |
| Information sources | 6 | Specify all databases, registers, websites, organisations, reference lists and other sources searched or consulted to identify studies. Specify the date when each source was last searched or consulted. | Method, 2.2 Search Strategy |
| Search strategy | 7 | Present the full search strategies for all databases, registers and websites, including any filters and limits used. | Supplementary Table 1 |
| Selection process | 8 | Specify the methods used to decide whether a study met the inclusion criteria of the review, including how many reviewers screened each record and each report retrieved, whether they worked independently, and if applicable, details of automation tools used in the process. | Method, 2.3 Selection Process |
| Data collection process | 9 | Specify the methods used to collect data from reports, including how many reviewers collected data from each report, whether they worked independently, any processes for obtaining or confirming data from study investigators, and if applicable, details of automation tools used in the process. | Method, 2.4 Data Collection |
| Data items | 10a | List and define all outcomes for which data were sought. Specify whether all results that were compatible with each outcome domain in each study were sought (e.g. for all measures, time points, analyses), and if not, the methods used to decide which results to collect. | Method, 2.4 Data Collection |
|  | 10b | List and define all other variables for which data were sought (e.g. participant and intervention characteristics, funding sources). Describe any assumptions made about any missing or unclear information. | Method, 2.4 Data Collection |
| Study risk of bias assessment | 11 | Specify the methods used to assess risk of bias in the included studies, including details of the tool(s) used, how many reviewers assessed each study and whether they worked independently, and if applicable, details of automation tools used in the process. | Method, 2.5 Risk of Bias Assessment |
| Effect measures | 12 | Specify for each outcome the effect measure(s) (e.g. risk ratio, mean difference) used in the synthesis or presentation of results. | N/A |
| Synthesis methods | 13a | Describe the processes used to decide which studies were eligible for each synthesis (e.g. tabulating the study intervention characteristics and comparing against the planned groups for each synthesis (item #5)). | Methods, Sections 2.1 and 2.4; Results, Sections 3.2-3.4; Tables 1-3 |
|  | 13b | Describe any methods required to prepare the data for presentation or synthesis, such as handling of missing summary statistics, or data conversions. | N/A |
|  | 13c | Describe any methods used to tabulate or visually display results of individual studies and syntheses. | Results: Figure 2 PRISMA flow diagram; Tables 1-3; summary sections 3.2.7, 3.3.2, and 3.4.5. |
|  | 13d | Describe any methods used to synthesize results and provide a rationale for the choice(s). If meta-analysis was performed, describe the model(s), method(s) to identify the presence and extent of statistical heterogeneity, and software package(s) used. | Narrative synthesis presented in Results, Sections 3.2-3.4 and Discussion, Sections 4.1-4. |
|  | 13e | Describe any methods used to explore possible causes of heterogeneity among study results (e.g. subgroup analysis, meta-regression). | N/A |
|  | 13f | Describe any sensitivity analyses conducted to assess robustness of the synthesized results. | N/A |
| Reporting bias assessment | 14 | Describe any methods used to assess risk of bias due to missing results in a synthesis (arising from reporting biases). | Not reported |
| Certainty assessment | 15 | Describe any methods used to assess certainty (or confidence) in the body of evidence for an outcome. | Not reported |
| **RESULTS** | | |  |
| Study selection | 16a | Describe the results of the search and selection process, from the number of records identified in the search to the number of studies included in the review, ideally using a flow diagram. | Results, Section 3.1 Search Results; Figure 2 PRISMA flow diagram |
|  | 16b | Cite studies that might appear to meet the inclusion criteria, but which were excluded, and explain why they were excluded. | Results, Section 3.1 reports exclusion categories and counts; individual excluded studies are not cited. |
| Study characteristics | 17 | Cite each included study and present its characteristics. | Results, Table 1-3 |
| Risk of bias in studies | 18 | Present assessments of risk of bias for each included study. | Supplementary material, Risk of Bias |
| Results of individual studies | 19 | For all outcomes, present, for each study: (a) summary statistics for each group (where appropriate) and (b) an effect estimate and its precision (e.g. confidence/credible interval), ideally using structured tables or plots. | Results, Sections 3.2-3.4; Tables 1-3 present outcomes using direction of effect |
| Results of syntheses | 20a | For each synthesis, briefly summarise the characteristics and risk of bias among contributing studies. | Supplementary material, Risk of Bias |
|  | 20b | Present results of all statistical syntheses conducted. If meta-analysis was done, present for each the summary estimate and its precision (e.g. confidence/credible interval) and measures of statistical heterogeneity. If comparing groups, describe the direction of the effect. | N/A |
|  | 20c | Present results of all investigations of possible causes of heterogeneity among study results. | N/A |
|  | 20d | Present results of all sensitivity analyses conducted to assess the robustness of the synthesized results. | N/A |
| Reporting biases | 21 | Present assessments of risk of bias due to missing results (arising from reporting biases) for each synthesis assessed. | Supplementary material, Risk of Bias |
| Certainty of evidence | 22 | Present assessments of certainty (or confidence) in the body of evidence for each outcome assessed. | Not reported |
| **DISCUSSION** | | |  |
| Discussion | 23a | Provide a general interpretation of the results in the context of other evidence. | Discussion, Sections 4, 4.1, and 4.2. |
|  | 23b | Discuss any limitations of the evidence included in the review. | Discussion, Section 4.3 Limitations |
|  | 23c | Discuss any limitations of the review processes used. | Not reported |
|  | 23d | Discuss implications of the results for practice, policy, and future research. | Discussion, Section 4.2 and Conclusion, |
| **OTHER INFORMATION** | | |  |
| Registration and protocol | 24a | Provide registration information for the review, including register name and registration number, or state that the review was not registered. | Methods, Section 2: PROSPERO registration CRD420251265631. |
|  | 24b | Indicate where the review protocol can be accessed, or state that a protocol was not prepared. | Not reported |
|  | 24c | Describe and explain any amendments to information provided at registration or in the protocol. | PROSPERO registration CRD420251265631 |
| Support | 25 | Describe sources of financial or non-financial support for the review, and the role of the funders or sponsors in the review. | Title page |
| Competing interests | 26 | Declare any competing interests of review authors. | Title page |
| Availability of data, code and other materials | 27 | Report which of the following are publicly available and where they can be found: template data collection forms; data extracted from included studies; data used for all analyses; analytic code; any other materials used in the review. | Available materials and data will be included in supplementary material |

*From:*  Page MJ, McKenzie JE, Bossuyt PM, Boutron I, Hoffmann TC, Mulrow CD, et al. The PRISMA 2020 statement: an updated guideline for reporting systematic reviews. BMJ 2021;372:n71. doi: 10.1136/bmj.n71

Supplementary Table 3. Risk of bias assessment for cell-based studies

| **Study** | **Selection Bias** | | **Performance Bias** | | **Detection Bias** | | **Attrition Bias** | | **Reporting Bias** | | **Other Bias** | |
| --- | --- | --- | --- | --- | --- | --- | --- | --- | --- | --- | --- | --- |
| Galbiati et al. 2002 | Low/Unclear | The sources of the cells are clearly stated, but authentication/passage is missing. | Low | The study used standardized culture conditions and vehicle control for all calcitriol treatments. | Unclear | There is no statement about blinding of the researchers when analysing western blot bands or counting TUNEL assays. | Low | The results had all cell lines mentioned. There is no statement of missing or excluded data. | Low | The researchers reported on all primary outcomes mentioned in their methods. | Low | The study was funded by a mix of academic and non-profit sources, and the authors' affiliations with Roche are disclosed. |
| Riachy et al. 2002 | Low | RINm5F source is cited, but authentication/passage is missing. Details on human islet isolation and donor characteristics are provided. | Low | All cytokine treatments were compared against identical vehicle controls, and experimental timing was strictly controlled. | Unclear | There is no statement about blinding of the researchers during the manual Hoechst staining analysis. | Low | The results had all cell lines mentioned. There is no statement of missing or excluded data. | Low | The researchers reported on all primary outcomes mentioned in their methods. | Low | Funding was provided by public and non-profit institutions and no conflicts of interest were identified. |
| Galbiati et al. 2003 | Low | The sources of the cells are clearly stated, and genetically modified cell lines are used to reduce uncertainty of the cell's identity. | Low | The study used standardized culture conditions and vehicle control for all calcitriol treatments. | Unclear | There is no statement about blinding of the researchers when analysing western blots or the DNA laddering assays. | Low | The results had all cell lines mentioned. There is no statement of missing or excluded data. | Low | The researchers reported on all primary outcomes mentioned in their methods. | Low | The study was funded by a mix of academic and non-profit sources, and no conflicts of interest were identified. |
| Riachy et al. 2006 | Low | The study used islets from 11 different donors, and islets were standardized by size. | Low | The study used standardized culture conditions and vehicle control for all calcitriol treatments. | Low | Most outcomes were measured using highly objective tools (FACS, RT-qPCR, cDNA Microarrays). | Low | The results had all cell lines mentioned. There is no statement of missing or excluded data. | Low | The researchers reported on all primary outcomes mentioned in their methods. | Low | The study was funded by academic research institutions, and no conflicts of interest were identified. |
| Wolden-Kirk et al. 2014 | Low | The sources of the cells are clearly stated, but specific randomization to groups was not detailed. | Low | The study used standardized culture conditions and vehicle control for all calcitriol treatments. | Low | Most outcomes were measured using highly objective tools (Affymetrix microarrays, ELISAs). Cell death was determined by two observers with one being blinded. | Low | The results had all cell lines mentioned. There is no statement of missing or excluded data. | Low | The researchers reported on all primary outcomes mentioned in their methods. | Low | The study was funded by academic and regional grants, and no conflicts of interest were identified. |
| Yang et al. 2015 | Low | The study used INS-1 cells, a stable and standardized rat insulinoma cell line. The researchers also ensured all cells were at the same passage. | Low | The study used standardized culture conditions and vehicle control for all calcitriol treatments. | Low | Most outcomes were measured using highly objective tools (Flow cytometry, western blotting, RT-qPCR). | Low | The study presents data for all three tested concentrations of vitamin D, and no statement of missing or excluded data. | Low | The researchers reported both the successful protection at higher doses and the lack of significance at lower doses of vitamin D. | Low | The study was funded by academic and regional grants, and no conflicts of interest were identified. |
| Wang et al. 2016 | Low | The Study used MIN6 cells, a stable and standardized mouse insulinoma cell line. | Low | The study used standardized culture conditions and vehicle control for all calcitriol treatments. | Low | Most outcomes were measured using highly objective tools (Flow cytometry, western blotting, RT-qPCR). | Low | The results had all cell lines mentioned. There is no statement of missing or excluded data. | Low | The researchers reported on all primary outcomes mentioned in their methods. | Low | The study was funded by academic sources, and no conflicts of interest were identified. |
| Wei et al. 2018 | Low | The researchers used stable and standardized cell lines (INS-1) and iPSC-derived β-like cells. They also verified findings using primary islets from both mice and human donors. | Low | The study used standardized culture conditions and vehicle control for all calcitriol treatments. | Low | Most outcomes were measured using objective, automated tools (RNA-seq, ELISA, TEM) | Low | The results had all cell lines mentioned. There is no statement of missing or excluded data. | Low | The researchers reported on all primary outcomes mentioned in their methods. | Low | The study was funded by a mix of academic and non-profit sources, and no conflicts of interest were identified. |
| Chen et al. 2019 | Low | The Study used EndoC-βH1 cells, a stable and standardized mouse insulinoma cell line. | Low | The use of transfections and siRNA ensures that the results were due to specific gene changes (VDR/FoxO1). | Low | Outcomes were measured using highly objective, automated tools (Flow cytometry, RT-qPCR, JC-1 dye). | Low | The study presents data for all intervention of vit D/VDR, and no statement of missing or excluded data. | Low | The researchers reported on all primary outcomes mentioned in their methods. | Low | The study was funded by academic and regional grants, and no conflicts of interest were identified. |
| He et al. 2019 | Low | The study used INS-1 cells, a stable and standardized rat insulinoma cell line. | Low | The study utilized a vehicle control, and the researchers ensured that control cells were treated with the same medium and conditions. | Low | Outcomes were measured using highly objective, automated tools (Flow cytometry, ELISA, western blotting, CCK-8 assay). | Low | The study had no statement of missing or excluded data. | Low | The researchers reported on all primary outcomes mentioned in their methods. | Low | The study was funded by academic and regional grants, and no conflicts of interest were identified. |
| Deng et al. 2019 | Low | The researchers utilized the NIT-1 cell line, which is a standardized pancreatic beta-cell line derived from transgenic mice | Low | The experiment was standardized. The cells were all cultured in the same medium (F12K) and kept in the same environment. | Low | Outcomes were measured using highly objective, automated tools (Flow cytometry, ELISA, western blotting, CCK-8 assay). | Low | The study had no statement of missing or excluded data. | Low | The researchers reported on all primary outcomes mentioned in their methods. | Low | The study was funded by academic sources and regional hospital, and no conflicts of interest were identified. |
| Bornstedt et al. 2020 | Low | The study used INS-1E cells, a stable and standardized rat insulinoma cell line. | Low | The experiment was standardized. All cells were maintained in the same medium. The study compared 1,25(OH)₂D and 25(OH)D at the same concentration and for the same duration against a control group. | Low | Outcomes were measured using highly objective, automated tools (Affymetrix GeneChip Microarray, ELISA, Bioinformatics). | Low | The study had no statement of missing or excluded data. | Low | The researchers reported all the gene changes, Including both upregulated and downregulated genes. | Low | The study was funded by internal academic and hospital grants, and no conflicts of interest were identified. |
| Hu et al. 2021 | Low | The study used MIN6 cells, a stable and standardized mouse insulinoma cell line. | Low | The study used standardized culture conditions and vehicle control for all calcitriol treatments. | Low | Outcomes were measured using highly objective, quantitative assays (Flow cytometry, western blotting, CCK-8 essay). | Low | The study performed all experiments in triplicate. There is no statement of missing or excluded data. | Low | The researchers reported on all primary outcomes mentioned in their methods. | Low | The study was funded government and academic grants, and no conflicts of interest were identified. |
| Wu et al. 2022 | Low | The study used INS-1E cells, a stable and standardized rat insulinoma cell line. | Low | The study used standardized culture conditions. The protocols for Vitamin D treatment and the siRNA transfection for AMPK knockdown were described with high precision. | Low | Outcomes were measured using highly objective, quantitative assays (Flow cytometry, western blotting, GSIS, PI staining). | Low | The study performed all experiments in triplicate. There is no statement of missing or excluded data. | Low | The authors reported a full mechanistic chain via AMPK, confirming by AMPK siRNA. They did not just show that vitamin D works. | Low | This research was funded by government and academic grants, and no conflicts of interest were identified. |
| Zhao et al. 2022 | Low | The study used INS-1 cells, a stable and standardized rat insulinoma cell line. | Low | The study used standardized culture conditions and vehicle control for all calcitriol treatments. | Low | Outcomes were measured using highly objective, quantitative assays (Western blotting, ELISA, chemical assays). | Low | The study performed all experiments in triplicate. There is no statement of missing or excluded data. | Low | They provided a complete picture of the pathway rather than just reporting favourable isolated results. | Low | This research was funded by academic grants, and no conflicts of interest were identified. |
| Ding & Wu,2022 | Low | The study used INS-1 cells, a stable and standardized rat insulinoma cell line. | Low | The study used standardized culture conditions and vehicle control for all calcitriol treatments. | Low | Outcomes were measured using highly objective, quantitative assays (Western blotting, ELISA, CCK-8 assay, iron assay kit). | Low | The study had no statement of missing or excluded data. | Low | The researchers reported on all primary outcomes mentioned in their methods. | Low | This research was funded by government and academic grants, and no conflicts of interest were identified. |
| Liu, Zhang, et al. 2023 | Low | The study used MIN6 cells, a stable and standardized mouse insulinoma cell line. | Low | The study used standardized culture conditions and vehicle control for all calcitriol treatments. | Low | Outcomes were measured using highly objective, quantitative assays (ELISA, TUNEL, CCK-8). | Low | The study had no statement of missing or excluded data. | Low | The researchers reported on all primary outcomes mentioned in their methods. | Low | This research was funded by hospital and academic grants, and no conflicts of interest were identified. |
| Zheng et al. 2025 | Low | The study used MIN6 cells, a stable and standardized mouse insulinoma cell line. | Low | The study used standardized culture conditions and established an optimal dose for vitamin D prior to the main experiments. | Low | Outcomes were measured using highly objective, quantitative assays (ELISA, Western blot, LDH assay). | Low | The study had no statement of missing or excluded data. | Low | The researchers reported on all primary outcomes mentioned in their methods. | Low | This research was funded by academic grants, and no conflicts of interest were identified. |

Supplementary Table 4. Risk of bias assessment for animal-based studies

| **Study** | **Selection Bias** | | **Performance Bias** | | **Detection Bias** | | **Attrition Bias** | | **Reporting Bias** | | **Other Bias** | |
| --- | --- | --- | --- | --- | --- | --- | --- | --- | --- | --- | --- | --- |
| Galbiati et al. 2002 | Moderate | The study states mice were randomly assigned to groups, but it does not describe the specific method of sequence generation or if allocation was blinded from researchers. | Unclear | There is no mention of random housing, and if the researchers administering the calcitriol injections were blinded to the treatment groups. | Low | The morphometric analysis and histology are quantitative and objective, but no statement about blinding of the researchers. | Low | There is no indication of "missing" animals or unexplained deaths during the study period. | Low | The researchers reported on all primary outcomes mentioned in their methods. | Low | The study was funded by a mix of academic and non-profit sources, and the authors' affiliations with Roche are transparently disclosed on the front page. |
| Gysemans et al. 2005 | Low | The study utilized NOD mice, which are the standardized genetic model for T1D. They also age and gender matched littermates and randomized into treatment groups. | Unclear | There is no mention of random housing, and if the researchers administering the calcitriol were blinded to the treatment groups. | Low | Outcome assessment (blood glucose levels, mRNA) was largely objective, but no statement about blinding of the researchers. | Low | There is no indication of "missing" animals or unexplained deaths during the study period. | Low | The researchers reported on all primary outcomes mentioned in their methods. | Low | The study was funded by academic sources, and no conflicts of interest were identified. |
| Kiekhaefer et al. 2011 | Low | The researchers used a combination of alternating and random assignment for the mice. | Unclear | There is no mention of random housing, and if the researchers were blinded to which mouse received the 2AMD or the oil. | Low | The blood glucose levels assessment, morphometric analysis and histology are quantitative and objective, but no statement about blinding of the researchers. | Low | There is no indication of "missing" animals or unexplained deaths during the study period. | Low | The researchers reported both successes and failures, and all other outcomes mentioned in their methods. | Low | The study was funded by academic sources, and no conflicts of interest were identified. |
| Wang et al. 2016 | Moderate | The study used a single strain, age and sex BL/6 mice. Specific method of randomization wasn't detailed, but treatment groups was clearly defined with consistent starting weight. | Unclear | There is no mention of random housing, and if the researchers administering the calcitriol were blinded to the treatment groups. | Moderate | Blood glucose and serum insulin levels were objective, but the histopathological analysis of the islets was a visual assessment. The study does not state if the researchers were blinded. | Low | There is no indication of "missing" animals or unexplained deaths during the study period. | Low | The researchers reported on all primary outcomes mentioned in their methods. | Low | The study was funded by academic sources, and no conflicts of interest were identified. |
| Su et al. 2017 | Moderate | The researchers stated that the rats were randomized into three groups based on body weight, but specific method of randomization wasn't detailed. | Unclear | There is no mention of random housing, and if the researchers administering the calcitriol were blinded to the treatment groups. | Moderate | RT-qPCR, ELISA, and radioimmunoassay were objective, but islet morphology analysis was visual, the study does not state if the researchers were blinded. | Low | There is no indication of "missing" animals or unexplained deaths during the study period. | Low | The researchers reported on all primary outcomes mentioned in their methods. | Low | The study was funded by academic and regional grants, and no conflicts of interest were identified. |
| Wei et al. 2018 | Moderate | The study used standardized, commercially available genetic mice strains. Specific method of randomization wasn't detailed, but they made sure that the mice were age-matched littermates. | Unclear | There is no mention of random housing, and if the researchers administering the calcitriol were blinded to the treatment groups. | Moderate | While glucose monitoring and RT-qPCR were objective, but the study did not state if the researchers performing the cell counting were blinded to the treatment group. | Low | There is no indication of "missing" animals or unexplained deaths during the study period. | Low | The researchers reported on all primary outcomes mentioned in their methods. | Low | The study was funded by a mix of academic and non-profit sources, and no conflicts of interest were identified. |
| Morró et al. 2020 | Low | The study utilized genetically defined mouse strains. The transgenic mice were compared against age-matched Wild-Type littermates. | Moderate | There is no mention of random housing. No Vit D intervention was needed as the mice was genetically modified to increase VDR expression. | Low | Outcome measures were highly objective and machine quantified. Blood glucose, protein and mRNA levels via Western Blot, ELISA, and RT-qPCR, and researchers used automated or semi-automated morphometric analysis for histology. | Low | There is no indication of "missing" animals or unexplained deaths during the study period. | Low | The researchers reported on all primary outcomes mentioned in their methods. | Low | This research was funded by public and academic grants, and no conflicts of interest were identified. |
| Lai et al. 2021 | Low | The study utilized NOD mice, which are the standardized genetic model for T1D. They also age-matched littermates and randomized into treatment groups. | Unclear | There is no mention of random housing, and if the researchers supplementing vitamin D diet were blinded to the treatment groups. | Moderate | The study used highly objective, quantitative tools (Flow Cytometry, ELISA, and TUNEL staining). However, it does not state that the researchers performing the histological scoring (H&E and TUNEL) were blinded to the treatment groups. | Low | There is no indication of "missing" animals or unexplained deaths during the study period. | Low | The researchers reported on all primary outcomes mentioned in their methods. | Low | The study was funded by academic and regional grants, and no conflicts of interest were identified. |
| Fathi et al. 2022 | Moderate | The researchers utilized 40 adult male Wistar rats of similar weight and age. The rats were randomly assigned into four equal groups. But it does not describe the specific method of sequence generation or if allocation was blinded from researchers. | Unclear | There is no mention of random housing, and if the researchers administering the calcitriol were blinded to the treatment groups. | Low | Outcome measures were highly objective and machine-quantified: ELISA, spectrophotometry, RT-qPCR. | Low | There is no indication of "missing" animals or unexplained deaths during the study period. | Low | The researchers reported on all primary outcomes mentioned in their methods. | Low | This research was funded by academic grants, and no conflicts of interest were identified. |
| Wu et al. 2022 | Moderate | The study states that 5-week-old male SD rats were randomly assigned into three groups. but it does not describe the specific method of sequence generation or if allocation was blinded from researchers. | Unclear | There is no mention of random housing, and if the researchers administering the calcitriol were blinded to the treatment groups. | Moderate | The study not explicitly mentioned if the researchers were blinded during the assessment of outcomes, such as reading the histological slides or calculating the IPGTT results. | Low | There is no indication of "missing" animals or unexplained deaths during the study period. | Low | The researchers reported on all primary outcomes mentioned in their methods. | Low | This research was funded by government and academic grants, and no conflicts of interest were identified. |
| Zhao et al. 2022 | Moderate | The study utilized ZDF rats, which are the standardized genetic model for T2D. The rats were randomly assigned, but it does not describe the specific method of sequence generation or if allocation was blinded from researchers. | Unclear | There is no mention of random housing, and if the researchers administering the calcitriol were blinded to the treatment groups. | Moderate | The histological assessment of islet morphology is somewhat subjective, and the paper does not mention if the pathologist was blinded to the treatment groups. | Low | There is no indication of "missing" animals or unexplained deaths during the study period. | Low | The researchers reported on all primary outcomes mentioned in their methods. | Low | This research was funded by academic grants, and no conflicts of interest were identified. |
| Ding & Wu,2022 | Moderate | The study utilized SD rats, and they were randomly assigned, but it does not describe the specific method of sequence generation or if allocation was blinded from researchers. | Unclear | There is no mention of random housing, and if the researchers administering the calcitriol were blinded to the treatment groups. | Low | The primary outcomes were highly objective and measured using automated equipment (ELISA, glucose meter, OGTT). | Low | There is no indication of "missing" animals or unexplained deaths during the study period. | Low | The researchers reported on all primary outcomes mentioned in their methods. | Low | This research was funded by government and academic grants, and no conflicts of interest were identified. |
| Liu et al. 2023 | Unclear | The study stated that the mice were divided into groups but does not specify how they were divided and if allocation was blinded from researchers. | Unclear | There is no mention of random housing, and if the researchers administering the STZ injection or the plasmid transfections were blinded to the treatment groups. | Low | The primary outcomes were highly objective and measured using automated equipment (ELISA, glucose meter, Western blot, qPCR). | Low | There is no indication of "missing" animals or unexplained deaths during the study period. | Low | The researchers reported on all primary outcomes mentioned in their methods. | Low | This research was funded by hospital and academic grants, and no conflicts of interest were identified. |
| Liu, Zhang, et al. 2023 | Moderate | The study stated that the mice were randomly divided into groups, but does not specify how they were divided and if allocation was blinded from researchers. | Unclear | There is no mention of random housing, and if the researchers administering the calcitriol were blinded to the treatment groups. | Moderate | The histological assessment of islet morphology is somewhat subjective, and the paper does not mention if the pathologist was blinded to the treatment groups. | Low | There is no indication of "missing" animals or unexplained deaths during the study period. | Low | The researchers reported on all primary outcomes mentioned in their methods. | Low | This research was funded by hospital and academic grants, and no conflicts of interest were identified. |
| Jia et al. 2024 | Moderate | The study utilized SD rats, and they were randomly assigned, but it does not describe the specific method of sequence generation or if allocation was blinded from researchers. | Unclear | There is no mention of random housing, and if the researchers administering the vitamin D3 and glibenclamide were blinded to the treatment groups. | Low | The primary outcomes were highly objective and measured using automated equipment (ELISA, glucose meter, TUNEL assay, qPCR). | Low | There is no indication of "missing" animals or unexplained deaths during the study period. | Low | The researchers reported on all primary outcomes mentioned in their methods. | Low | This research was funded by academic grants, and no conflicts of interest were identified. |
| Zheng et al. 2025 | Moderate | The study stated that the mice were randomly divided into groups, but does not specify how they were divided and if allocation was blinded from researchers. | Unclear | There is no mention of random housing, and if the researchers administering the vitamin D3 and STZ were blinded to the treatment groups. | Low | The primary outcomes were highly objective and measured using automated equipment (ELISA, glucose meter, Western blot, qPCR). | Low | There is no indication of "missing" animals or unexplained deaths during the study period. | Low | The researchers reported on all primary outcomes mentioned in their methods. | Low | This research was funded by academic grants, and no conflicts of interest were identified. |
